# CellCoal: Coalescent Simulation of Single-Cell Sequencing Samples

**DOI:** 10.1093/molbev/msaa025

**Published:** 2020-02-06

**Authors:** David Posada

**Affiliations:** m1 Department of Biochemistry, Genetics and Immunology, University of Vigo, Vigo, Spain; m2 Biomedical Research Center (CINBIO), University of Vigo, Vigo, Spain; m3 Galicia Sur Health Research Institute, Vigo, Spain

**Keywords:** somatic evolution, single-cell genomics, allele dropout, amplification error

## Abstract

Our capacity to study individual cells has enabled a new level of resolution for understanding complex biological systems such as multicellular organisms or microbial communities. Not surprisingly, several methods have been developed in recent years with a formidable potential to investigate the somatic evolution of single cells in both healthy and pathological tissues. However, single-cell sequencing data can be quite noisy due to different technical biases, so inferences resulting from these new methods need to be carefully contrasted. Here, I introduce CellCoal, a software tool for the coalescent simulation of single-cell sequencing genotypes. CellCoal simulates the history of single-cell samples obtained from somatic cell populations with different demographic histories and produces single-nucleotide variants under a variety of mutation models, sequencing read counts, and genotype likelihoods, considering allelic imbalance, allelic dropout, amplification, and sequencing errors, typical of this type of data. CellCoal is a flexible tool that can be used to understand the implications of different somatic evolutionary processes at the single-cell level, and to benchmark dedicated bioinformatic tools for the analysis of single-cell sequencing data. CellCoal is available at https://github.com/dapogon/cellcoal.

## Introduction

Most research in evolutionary biology has focused on the changes that occur in the germline across generations, within and between species. Much less attention has been paid to the process of change among the cells of a single individual, or somatic evolution. This has recently started to change, prompted by the advent of single-cell genomic techniques that allow the dissection of mixed cell populations in healthy and diseased tissues, providing the ultimate level of genomic resolution (Marioni and Arendt 2017; Tanay and Regev 2017). Indeed, single-cell genomics is expected to result in a major breakthrough not only in medical research but also in the study of a plethora of uncultured unicellular organisms that dominate many environments on earth (Woyke et al. 2017). So far, single-cell genomics has had an enormous impact in different biological fields, including neurobiology, development, microbiology, immunology, or cancer research (Wang and Navin 2015; Gawad et al. 2016; Wang and Song 2017; Ren et al. 2018).

However, the single-cell sequencing pipeline is not straightforward. In particular, to obtain the DNA sequence of a single cell it is necessary to amplify its genome first in order to have enough material for library construction—although library-free methods exist (Zahn et al. 2017), they have not been yet generalized. Unfortunately, single-cell whole-genome amplification (scWGA) entails several technical errors, such as nonuniform amplification of different genomic regions, which can ultimately lead to allelic imbalance (AI) and allelic dropout (ADO), generation of chimeric DNA molecules and amplification errors, due to the DNA polymerase strand displacement activity and infidelity (Lasken and Stockwell 2007; Voet et al. 2013; Navin 2014; Huang et al. 2015). These errors introduce several biases in the sequencing data, complicating the detection of structural and nonstructural variants. Most importantly for the purpose here, AI distorts the maternal and paternal read proportions, and in the case of ADO, true single-nucleotide variants (SNVs) can disappear from the data. In addition, amplification errors can induce false SNV calls.

Not surprisingly, dedicated SNV callers have been implemented for single-cell sequencing data (Zafar et al. 2016; Dong et al. 2017; Bohrson et al. 2019; Hård et al. 2019). At the same time, different tools have been developed for the reconstruction of phylogenetic trees (Subramanian and Schwartz 2015; Jahn et al. 2016; Ross and Markowetz 2016; Zafar et al. 2017) and genotypes (Singer et al. 2018; Zafar et al. 2019) from single-cell SNV data. Although these approaches have been benchmarked by the same authors using empirical data and computer simulations, these comparisons have been somewhat limited regarding the assessed scenarios, for example, with respect to demography, mutation model, or scWGA bias. Indeed, the systematic assessment of any software tool is both challenging and laborious (Mangul et al. 2019), but it can be facilitated by comprehensive, third-party simulation tools.

## New Approaches

**Fig. 1.**
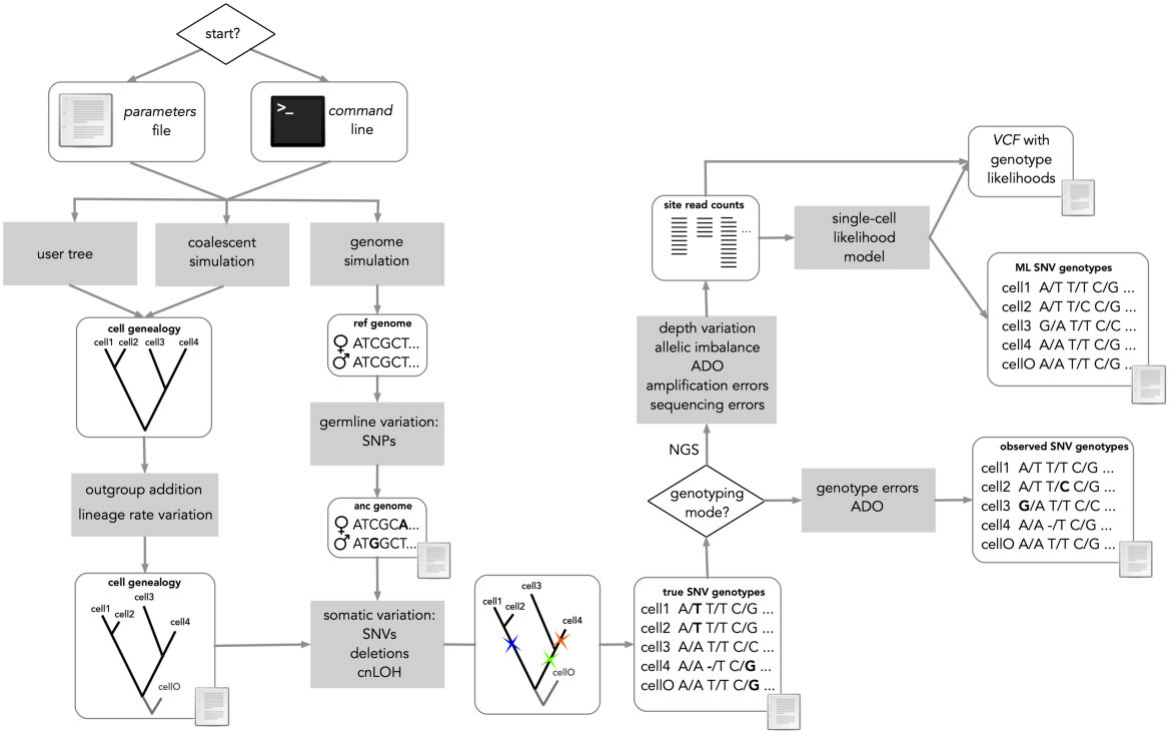
Main flow of CellCoal. First, a sample genealogy is simulated. Then, cell genotypes are evolved along this genealogy by introducing somatic mutations, deletions, and copy-neutral LOH. Finally, sequencing reads are produced considering the specific biases of single-cell sequencing.

Genetic simulations are playing an increasingly important role in evolutionary biology (Haller and Messer 2019). However, we currently lack a generic tool for the simulation of single-cell sequencing samples, and therefore for the assessment and comparison of methods for the evolutionary analysis of single-cell DNA data. In order to fill this gap, here I present CellCoal, a software tool for the simulation of single-cell sequencing genotypes obtained from cell populations. CellCoal works in three main steps (fig. 1). First, it generates a coalescent genealogy for a set of individual cells sampled from a given cell population growing under different demographic regimes. Second, it evolves diploid genotypes along this somatic genealogy under different mutation models, including SNVs, point deletions, or loss-of-heterozygosity events. Finally, it generates sequencing read counts and genotype likelihoods, considering technical artifacts such as AI, ADO, sequencing error, amplification error, or doublet cells, and outputs all the information to a VCF file. Below, I discuss the characteristics of CellCoal in more detail.

## Implementation Details

### Coalescent Genealogy

CellCoal starts by simulating a genealogy for the sampled cells under the neutral coalescent, going backward in time. Note that the coalescent assumes that the sampled cells come from a much larger population with constant or variable size. CellCoal implements a continuous exponential population growth model (Slatkin and Hudson 1991), with the option of multiple demographic periods (Hudson 2002), but also a specific parameterization of the coalescent for cancer cell samples (Ohtsuki and Innan 2017). The latter considers overlapping generations and the exponential growth results from the difference between cell birth and death rates. After the cell genealogy is simulated, two additional branches are added. First, a “root branch” is added joining the most recent common ancestor of the sample (sMRCA) with its most recent common ancestor with the outgroup (oMRCA). Second, an “outgroup branch” is added joining the oMRCA with the outgroup cell (e.g., a normal somatic cell as outgroup to a tumor cell phylogeny). The length of these two branches is controlled by the user. Among-lineage rate variation can be introduced using multipliers sampled from a gamma distribution. Biologically, this is interesting if we want to simulate (for example) a situation under which some lineages evolve at different rates due to changes in the somatic mutation rate, as seen, for example, in cancer cell populations (Podlaha et al. 2012).

### Somatic Genotype Evolution

CellCoal simulates the somatic evolution of cell genotypes along the coalescent genealogy—but the user can also specify its own tree—by adding single-nucleotide somatic mutations: SNVs, copy-neutral loss of heterozygosity events (cnLOH), and point deletions, starting from an ancestral genome (simulated or user-defined) at the oMRCA, in which germline variants can be added at a certain rate. Note that CellCoal does not simulate copy number alterations or structural variants. CellCoal considers two possible alphabets (binary or DNA) and several infinite and finite-site mutation models. Infinite-site models (ISM) (Kimura 1969) allow only one mutation per site. For DNA, 30 distinct trinucleotide mutational signatures (https://cancer.sanger.ac.uk/cosmic/signatures_v2) (*sensu*Alexandrov et al. 2013) can be simulated. These signatures represent the footprint of different mutational processes acting on human cells, and consist of the frequency of each mutational type (C**→**A, C**→**G, C**→**T, T**→**A, T**→**C, and T**→**G; all mutations are referred to by the pyrimidine of the mutated Watson–Crick base pair) considering the nucleotide context (bases immediately 5′ and 3′) in which they occur. For SNVs, CellCoal also implements several finite-site models (FSM), in which multiple mutations at a given site are possible. For binary data, the FSM implemented is known as Cavender–Farris–Neyman or Mk2 model (see Lewis 2001), and is equivalent to a Jukes–Cantor model (JC) (Jukes and Cantor 1969) for two alleles. For DNA data, reversible and nonreversible FSMs are possible, including popular substitution models such as JC, HKY (Hasegawa et al. 1985), or general-time-reversible (Tavaré 1986). In addition, mutation rates can vary among sites (Yang 1996). Finally, copy-neutral loss of heterozygosity (cnLOH) events (e.g., A/G **→** A/A, or A/G **→** G/G) can be added assuming a haploid ISM, which means that cnLOHs cannot happen in the same site twice unless they occur in a different maternal/paternal genome. Single-nucleotide deletions (e.g., N/N **→** –/N, or N/N **→** N/–) can also be added assuming a haploid ISM.

### Simulation of Single-Cell Genomics Noise

One of the main novelties of CellCoal is that it can simulate technical artifacts resulting from cell sorting, such as the presence of two cells in a sequencing library (i.e., “doublets”), or induced by scWGA, such as AI, ADO, or amplification errors. On top of these, it can produce sequencing errors at a given rate. ADO is introduced by choosing for each cell whether a given allele is amplified or not according to a specific probability. This probability can be constant, or vary across cells and/or sites according to a beta binomial distribution parameterized by the user.

Genotype errors due to the scWGA biases can be introduced in two distinct ways. In the simplest approach, genotype errors are directly imposed on the evolved genotypes. For DNA models, errors can be introduced with distinct probabilities according to a 4 × 4 matrix. In this case, genotype errors will be encapsulated into a single class representing different sources of error that can be introduced along the single-cell sequencing pipeline, including amplification, sequencing, and/or variant calling errors. Alternatively, CellCoal can generate independent read counts for each site given according to a Poisson distribution, with a mean sequencing coverage (depth) specified by the user. For a more heterogeneous, dispersed coverage, the user can specify a negative binomial distribution. To control for AI, at each site reads can be randomly assigned to the maternal or paternal allele according to a beta binomial distribution, but always conditioned on the particular ADO status of the site. Moreover, the user can control the reduction of the sequencing coverage at haploid sites (resulting from ADO) in comparison with diploid sites, which by default is 50%. All these factors, ADO, AI, read distribution across sites, and ploidy will contribute to the resulting nonuniformity of the coverage.

**Fig. 2.**
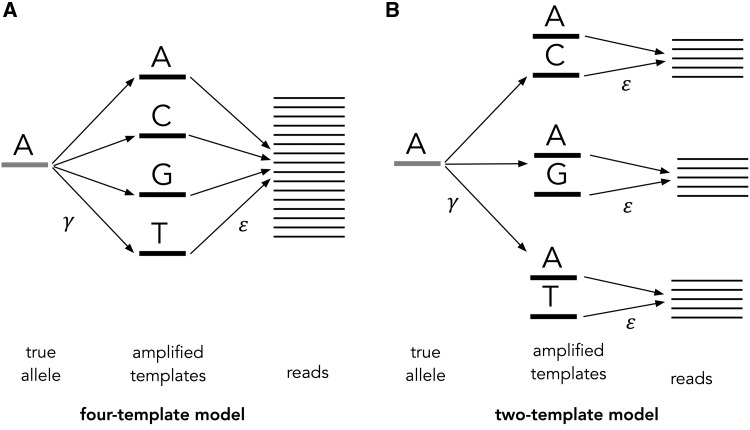
Amplification error models. Four-template (*A*) and two-template (*B*) model for amplification (γ) and sequencing (ε) error.

During the simulation of the read counts, amplification and sequencing errors are also introduced. The probability of an amplification error for a given site follows a beta binomial distribution with mean and variance specified by the user. The probability of the different types of errors (e.g., A**→**C, A**→**G, and A**→**T) can be specified in a 4 × 4 error matrix. CellCoal implements two novel amplification error models, depending on whether all four bases or just two, are allowed to be present in the set of amplified templates (fig. 2). Doublets are generated by mixing the read counts from two of the sampled single cells according to a beta binomial probability.

Once the read counts are in place, genotype calling is performed under different maximum likelihood models explained below.

### Single-Cell Genotype Likelihoods

CellCoal can calculate the likelihood of any genotype given the read counts simulated and the sequencing and amplification errors specified (see Korneliussen et al. 2013). This calculation allows, for example, for the identification of the maximum likelihood genotypes. The basic model used to calculate the genotype likelihoods, *Pr(D|G)*, is very similar to that implemented in GATK (McKenna et al. 2010; DePristo et al. 2011; Korneliussen et al. 2013):

$$\mathrm{Pr}\left(D|G=\{A_{1},A_{2}\}\right)=\prod_{i=1}^{M}\mathrm{Pr}\left(b_{i}|G=\{A_{1},A_{2}\}\right)=\prod_{i=1}^{M}\left(\frac{1}{2}\;p\left(b_{i}|A_{1}\right)+\frac{1}{2}\;p\left(b_{i}|A_{2}\right)\right),$$

where *D* are the read counts, *G* is the genotype, *A* is the allele, *M* is the number of reads, and *b_i_* is the observed nucleotide base in read *i*. Moreover, it is straightforward to include ADO in this computation, as in Zafar et al. (2016):

$$\mathrm{Pr}\left(D\text{|}G=\{A_{1},A_{2}\}\right)=\left(1-\delta \right)\overset{M}{\underset{i=1}{\prod }}\mathrm{Pr}\left(b_{i}\text{|}G=\left\{A_{1},A_{2}\right\}\right)+\delta \left[\frac{1}{2}\overset{M}{\underset{i=1}{\prod }}\mathrm{Pr}\left(b_{i}\text{|}G=\left\{A_{1},–\right\}\right)+\frac{1}{2}\overset{M}{\underset{i=1}{\prod }}\mathrm{Pr}\left(b_{i}\text{|}G=\left\{–,A_{2}\right\}\right)\right]=\left(1-\delta \right)\overset{M}{\underset{i=1}{\prod }}\left(\frac{1}{2}p\left(b_{i}\text{|}A_{1}\right)+\frac{1}{2}p\left(b_{i}\text{|}A_{2}\right)\right)+\frac{\delta }{2}\overset{M}{\underset{i=1}{\prod }}p\left(b_{i}\text{|}A_{1}\right)+\frac{\delta }{2}\overset{M}{\underset{i=1}{\prod }}p(b_{i}\text{|}A_{2}),$$

where *δ* is the probability of ADO at a given site. These likelihoods can be calculated under three different error models: GATK-like, 4-template, and 2-template models, which only differ in the calculation of the probability of a particular read given the true allele, *p*(*b*|*A*).

#### GATK-Like Model

In the simplest case, we can assume the same sequencing error rate *ε* for all bases and sites, and no amplification error, so this probability becomes:

pbA=ε/3, b≠A1-ε, b=A,

where *ε* is the probability of sequencing error.

#### Four-Template Amplification Error Model

This model extends the previous one in order to consider amplification error, which, together with the sequencing error, can be different for distinct nucleotides. The amplification error in CellCoal is sampled for each site from a beta binomial distribution, as in Orton et al. (2015) and Zafar et al. (2017) for DNA polymerase and unspecified genotyping errors, respectively. This particular model allows for multiple amplification errors at a single site, and therefore, all four ACGT templates are possible (the correct one and the other three; fig. 2*A*). Here, *p*(*b*|*A*) takes the form:

$$p\left(b|A\right)=\sum_{j=1}^{4}p\left(t_{j}|A\right)\;p\left(b|t_{j}\right)=\sum_{j=1}^{4}\gamma_{A\to t_{j}}\varepsilon_{t_{j}\to b},$$

where

γi→j=γei→j, i≠j1-γ, i=j, εi→j=εei→j, i≠j1-ε, i=j

and where *t_j_* is the amplified template base (which can take four values corresponding to the four DNA nucleotides), γ is the probability of amplification error for a given site, $\gamma_{i\to j}$ is the probability of amplification of base *i* into template base *j*, $\varepsilon_{i\to j}$ is the probability of sequencing error from template base *i* to read base *j*, and $e_{i\to j}$ is the relative probability of amplification/sequencing error from base *i* to base *j*. Note that, if the amplification and sequencing errors are constant, this probability simplifies to:

pbA=1-γ ε/3+γ 1-ε/33, b≠A1-γ1-ε+γ ε/3, b=A.

#### Two-Template Amplification Error Model

This model is very similar to the previous one, but it assumes that only a single amplification error can occur at a single site, and that therefore only two templates (the “correct” and a "wrong" one) are possible (fig. 2*B*). In this case, *p*(*b*|*A*) is:

pbA=∑j=1j≠A4ptjA pbtj=∑j=1j≠A4eA→tj1-γεA→b+γ εtj→b.

## Basic Usage

CellCoal works on the Linux/Mac command line in a noninteractive fashion. CellCoal can parse its arguments directly from the command line, as in the following example:

cellcoal-x.y.z -n100 -s20 -l1000 -e10000 -g1.0e-04 -k1 -i1 -b1 -j250 -p0.0 -f0.3 0.2 0.2 0.3 -r0.00 0.03 0.12 0.04 0.11 0.00 0.02 0.68 0.68 0.02 0.00 0.11 0.04 0.12 0.03 0.00 -1 -2 -3 -4 -6 -9 -v -x -#200011

where *cellcoal-x.y.z* is the executable file, -*n* is the number of simulation replicates; -*s* is the number of sampled cells; *-l* is the number of sites; *-e* is the effective population size; *-g* is the population growth rate; -*k* is the root branch length ratio; -*i* is the amount of rate variation among lineages; -*b* is the alphabet (DNA in this case); -*j* is a fixed number of mutations; -*f* are the nucleotide frequencies; -*r* are the relative mutation rates among nucleotides; -*1*, -*2*, -*3*, -*4*, -*6*, -*9*, -*v*, and -*x* are different options controlling which type of information is printed to the output files; and -*#* is the random seed.

If no arguments are passed in the command line, CellCoal will look for a file called “parameters” in the directory of the binary file. The “parameters” file is a text file that contains the different arguments for the simulation. A brief usage guide plus the current default values for the simulation parameters can be obtained typing “*cellcoal* *-h*.” Detailed documentation and example scripts are available at https://github.com/dapogon/cellcoal.

## Example: Effect of the Sequencing Coverage Heterogeneity on Single-Cell Genotypes

It is well known that sequencing coverage can be very heterogenous for single-cell sequencing data (Navin 2014). To illustrate a potential use of CellCoal, I designed an experiment to study how sequencing coverage heterogeneity affects the quality of the genotypes inferred. I explored 12 scenarios consisting of four levels of coverage heterogeneity, times three different sequencing coverages or depths. Coverage heterogeneity followed a negative binomial distribution, with three different values for the mean (1×, 5×, 10×) and four for the dispersion parameter (1, 5, 10, infinite). Smaller dispersion values result in more coverage heterogeneity, and when the dispersion is infinite the negative binomial distribution becomes a Poisson. For each scenario, I simulated 100 samples, each with 100 cells and 100 genomic sites, obtained from a population with an effective size of 10,000 and a growth rate of 0.1, and with a fixed number of 100 mutations taking place along the sample genealogy according to an infinite-site diploid model. For simplicity, I set the relative lengths of the root and outgroup branches to 0, respectively, and there was no ADO, AI, or sequencing error. This simulation takes less than a minute in a standard personal laptop. I then compared the maximum likelihood genotypes obtained under the true generating model with the true genotypes, and computed the number of wrong genotypes inferred, plus the proportion of called genotypes and the total number of SNVs observed.

**Fig. 3.**
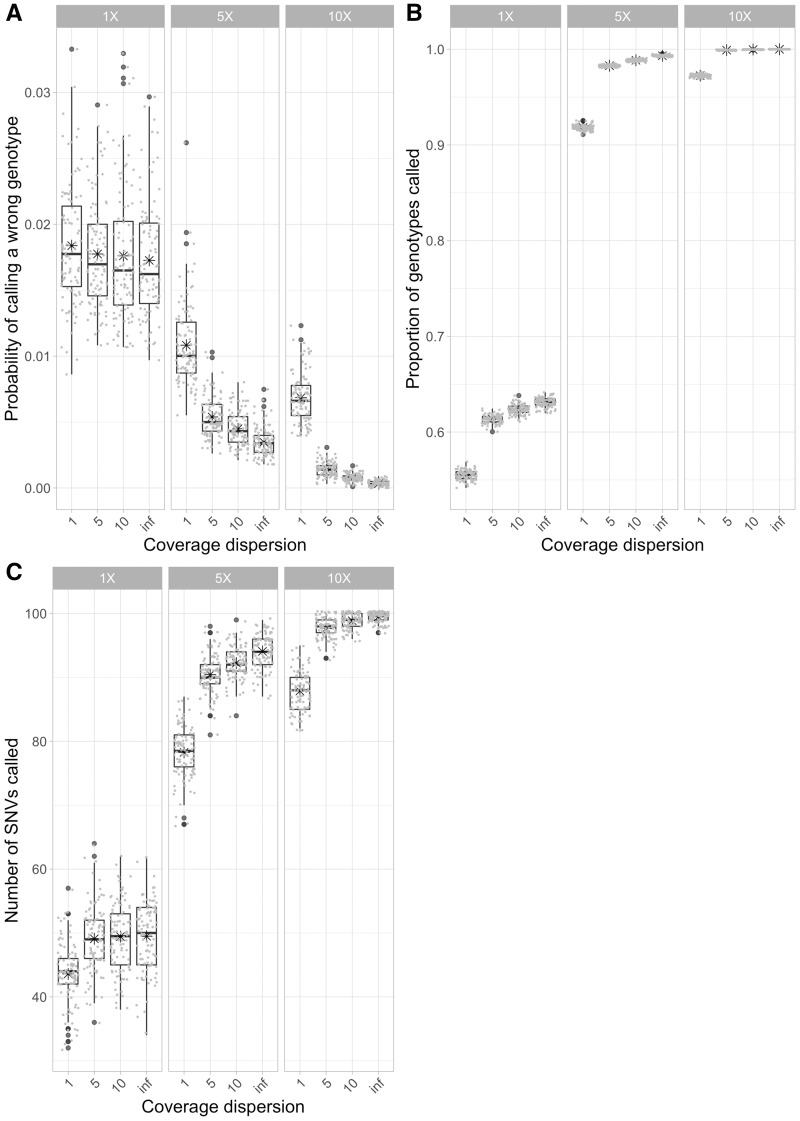
Effect of sequencing coverage heterogeneity on single-cell genotypes. (*A*) Probability that the maximum likelihood genotype is wrong. (*B*) Proportion of genotypes called. (*C*) Total number of single-nucleotide variants (SNVs) called. GATK and true (GATK+ADO) are the likelihood models used for calling genotypes. Coverage dispersion corresponds to the negative binomial dispersion parameter. The smaller this parameter is, the more heterogeneity there is. At the top, 1×, 5×, and 25× are different overall sequencing depths. In the boxplots, the central line indicates the median, whereas the box limits correspond to the Q1 and Q3 quartiles and the asterisk to the mean.

The results suggest that the level of coverage heterogeneity across sites has a detrimental effect on the accuracy of the inferred genotypes (fig. 3). Under the standard GATK likelihood model, more heterogeneity results in less accurate genotypes, particularly at low-sequencing depth. Note that the absolute value of the genotype error is not that relevant here, as it decreases with the number of cells in the sample because with more cells the proportion of homozygotes for the reference allele can only be higher. For example, if we simulate only ten cells, the genotype error increases five times. The amount of missing data and the number of SNVs observed—in this experiment, the true number is 100—depended mostly on the sequencing depth, but when coverage is most heterogenous (i.e., the dispersion parameter is 1), a noticeable amount of SNVs are missed. These results suggest that, in general, WGA kits that provide a more homogeneous coverage across sites are preferred, even when the sequencing depth is 10×, which can be considered already high for a single-cell considering the current costs for whole-genome sequencing. In addition, they indicate that increasing the coverage above 5× does not result in substantial improvements of the quality of the inferences, as we have already suggested for empircal data (Alves and Posada 2018). In the Supplementary Material online, I describe another two simulation experiments performed with CellCoal that explore the role of amplification error and ADO on single-cell genotype calls.

## Discussion

Somatic evolution has been ignored for decades, mostly because lack of technical tools for the assessment of genomic differences among the individual cells of a single organism. More recently, multiregional genomic studies in human cancers—by far, the most studied scenario of somatic evolution, at least in humans—and in healthy tissues have unveiled a large amount of somatic differences among the cells of different parts of our body (e.g., Lee-Six et al. 2018; Martincorena, Fowler, et al. 2018). Clearly, the advent of single-cell genomics has now opened the door for very detailed studies of somatic evolution in different tissues, pathological or not, and in multiple species (Dou et al. 2018; Lodato et al. 2018). It is therefore important to develop new methods for the analysis of single-cell data, and to benchmark them (e.g., using computer simulations). CellCoal is, as far as I know, the first available software specifically designed to simulate the evolution of single-cell samples together with the obtention of single-cell sequencing data, and one of its main uses will be to benchmark different aspects of the single-cell sequencing pipeline, from variant calling to populational and phylogenetic inference.

CellCoal is a flexible tool that tries to balance a trade-off between computational efficiency and realism at different levels: populational, genomic, and technical. In this regard, CellCoal is a fast tool able to consider the cell population demography, the genealogy of the sampled cells, different mutation models at the DNA level, and the effect of the biases inherent to single-cell genome amplification on the distribution of the sequencing read counts. Like any other simulator, CellCoal has limitations. In particular, the cell genealogies are sampled from the neutral coalescent, whereas in some specific scenarios, like the tumoral one, selection among somatic clones is thought to be quite relevant, at least during tumor establishment (Sottoriva et al. 2015; Martincorena, Raine, et al. 2018; Williams et al. 2018). However, several studies indicate that a great deal of sequence variation in tumors might be neutral (Ling et al. 2015; Williams et al. 2016; Niida et al. 2018; Tarabichi et al. 2018), suggesting that neutral evolution may be the most appropriate null model for comparison (Cannataro and Townsend 2018). Although selection has been modeled in the coalescent for specific, relatively simple, selective scenarios (Kaplan et al. 1988; Hey 1991; Neuhauser and Krone 1997), we currently lack a coalescent model for somatic clonal selection. Clearly, in selection-driven models, forward simulation rather than reverse-time coalescent models is preferable. Accordingly, several somatic forward simulators have been developed in the context of cancer (Diaz-Uriarte 2017; Iwasaki and Innan 2017; McDonald and Michor 2017), although without considering the specific biases of single-cell genomics. In CellCoal, one may introduce rate variation among branches, according to a gamma distribution, in an otherwise ultrametric coalescent genealogy, for example, to simulate a change in the mutation rate. Although such an approach might fit some scenarios resulting from weak selection (data not shown), to simulate data under selection one should preferably use instead as input a genealogy generated under a selective regime, for example, using one of the forward simulators mentioned above. In addition, CellCoal currently assumes that samples are taken from an unstructured population, whereas in some realistic scenarios obvious cell compartments might exist, like different cell types or cells from a primary tumor and distant metastases (e.g., Naxerova and Jain 2015; Arendt et al. 2016).

In CellCoal, the simulated sites are not necessarily spatially ordered, therefore, read counts are simulated independently for each site, therefore, without considering the correlation in coverage among physically close sites. A more realistic approach to simulate sequencing reads might be to introduce simulated mutations on real data sets, according to the genealogy (Ewing et al. 2015), but then one could not have fine control over the desired coverage homogeneity or the error level. Finally, CellCoal does not consider structural variants, focusing on mutational events that are detected at the single-nucleotide level. The main reason for this is that we lack solid statistical models for the somatic evolution of structural variants, at least nontrivial ones.

All in all, and despite its limitations, CellCoal should offer enough functionality for benchmarking single-cell sequencing strategies and tools, and for studying the implications of different evolutionary processes and technical errors at the single-cell level. CellCoal is free, and licensed under the GNU General Public License. It is available on GitHub (https://github.com/dapogon/cellcoal), together with documentation and example scripts. 

## Supplementary Material

Supplementary data are available at *Molecular Biology and Evolution* online.

## Supplementary Material

msaa025_Supplementary_Data
