# Supplementary material for: CellCoal: Coalescent Simulation of Single-Cell Sequencing Samples
Source: Mol Biol Evol. 2020 Feb 6;37(5):1535–42. doi: 10.1093/molbev/msaa025 (PMC7182211; doi:10.1093/molbev/msaa025)
Supplement: msaa025_Supplementary_Data [file msaa025_supplementary_data.pdf]

# Supplementary Material

# CellCoal: coalescent simulation of single-cell NGS genotypes

David Posada[1,2,3]

[1]Department of Biochemistry, Genetics and Immunology, University of Vigo, Spain.

[2]Biomedical Research Center (CINBIO), University of Vigo, Spain.

[3]Galicia Sur Health Research Institute, Vigo, Spain.

E-mail: dposada@uvigo.es

*Keywords*: somatic evolution, single-cell genomics, allele dropout, amplification error

## Supplementary Simulation Experiment 1: effect of the amplification error

The main purpose of this experiment was to investigate how amplification error affects the number of wrong genotypes inferred –both at variable and invariable sites in order to compare scenarios with a different number of variable sites– plus the proportion of called genotypes and the total number of SNVs called. Here I explored 24 scenarios, consisting of three levels of amplification error, times four different sequencing coverages, times two models for the calculation of genotype likelihoods. The amplification error probabilities followed a 4-template, beta binomial model, with three mean values (0, 0.001 and 0.01) and a constant variance of 0.0005. The sequencing coverage was sampled from a negative binomial distribution with four mean values (1X, 5X, 10X and 25X) and a constant dispersion of 5. The two likelihood models are described below. For each scenario, I simulated 100 samples (replicates) each with 100 cells and 200 genomic sites, obtained from a population with an effective size of 10000 and a growth rate of 0.1, with a fixed number of 100 mutations taking place along the sample genealogy according to an infinite-site diploid model. For simplicity, the relative lengths of the root and outgroup branches were set to 0, respectively, and there was no allelic dropout, allelic imbalance or sequencing error. The inferred genotypes correspond to CellCoal maximum likelihood estimates under the true generating model or enforcing the standard GATK model –which does not consider amplification error.

This experiment shows that the amplification error has a detrimental effect on the accuracy of the inferred genotypes, as expected (**fig. S1**). Under the true model, increasing the sequencing coverages in general reduces drastically the probability of wrong genotype calls (which increases if we simulate less cells; data not shown). However, under a standard GATK model (which is misspecified in the presence of amplification error), increasing the sequencing coverage has the opposite effect and exacerbates the proportion of wrong genotypes. Although the reported overall amplification errors during single-cell whole genome amplification tend to be low (<0.001), this experiment suggests that, in general, genotypes from single-cell sequencing data should always be inferred under models that consider the possibility of amplification error.

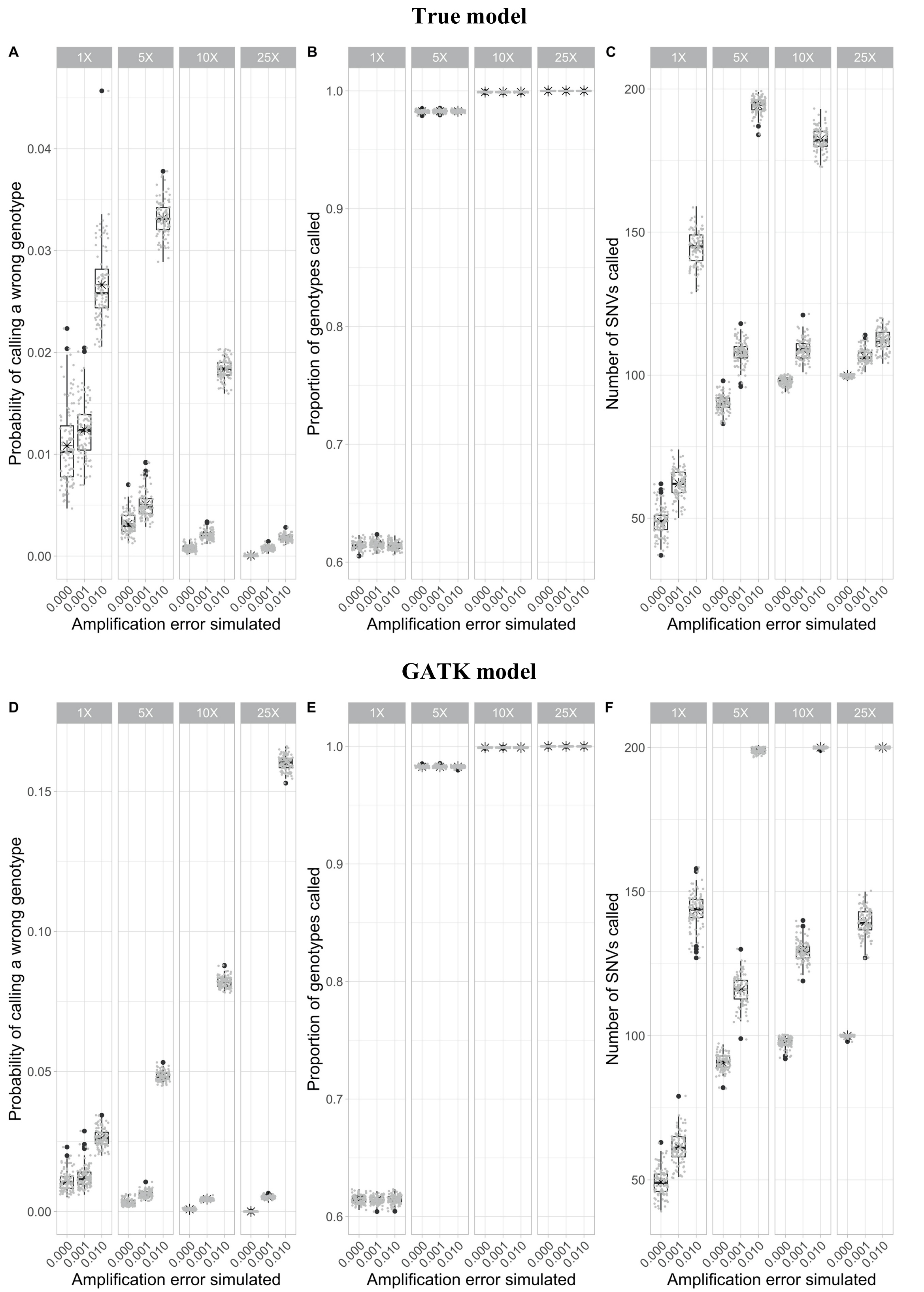


**FIG. S1**. Effect of single-cell amplification error on genotype calling across sequencing coverages. Panels A-C correspond to estimates under the true likelihood model, while D-F show results after enforcing the GATK model. *(A,D)* Probability that the maximum likelihood genotype is wrong, across variable and invariable sites. (*B,E*) Proportion of genotypes called. *(C,F)* Total number of single nucleotide variants (SNVs) called. 1X, 5X, 10X and 25X are the sequencing coverages. In the boxplots the central line indicates the median, while the box limits correspond to the Q1 and Q3 quartiles and the asterisk to the mean

## Supplementary Simulation Experiment 2: effect of allele dropout

This experiment is very similar to the previous experiment, but it was focused instead on the effect of allele dropout (ADO). In this case, I explored 18 scenarios consisting of three levels of ADO, times three different sequencing coverages, times two models for the calculation of genotype likelihoods. The ADO probabilities were constant across cells and sites, with three values (0, 0.3 and 0.6). The sequencing coverage was sampled from a Poisson distribution with three mean values (1X, 5X, and 25X) and a constant dispersion of 5. The two likelihood models explored for genotyping were the true model and the standard GATK model. For each scenario, I simulated 100 samples (replicates) each with 10 cells and 1000 genomic sites, obtained from a population with an effective size of 10000 and a growth rate of 0.1, with a fixed number of 1000 mutations taking place along the sample genealogy according to an infinite-site diploid model. For simplicity, the relative lengths of the root and outgroup branches were set to 0, respectively, and there was no amplification or sequencing error.

The probability of wrong genotype calls (which increases if we simulate less cells; data not shown) is highest overall –but at the same time less dependent on the ADO rate– at low sequencing coverages (1X) (**fig. S2**). At larger coverages the bias introduced by ADO is smaller, but clearly increases with the ADO rate. ADO decreases the proportion of genotypes called and reduces the observed number of SNVs. The results are very similar regarding of the consideration of ADO in the model (GATK vs the true model GATK+ADO).

ADO has a clear effect on the inference of single-cell genotypes, introducing many false negatives increasing missing data and decreasing the number of SNVs called. The inclusion of ADO parameter in the GATK model did not improve the inference, as the maximum likelihood genotype in the case of ADO is always the homozygote, despite the likelihood value of the heterozygote, which is higher when ADO is included in the model.

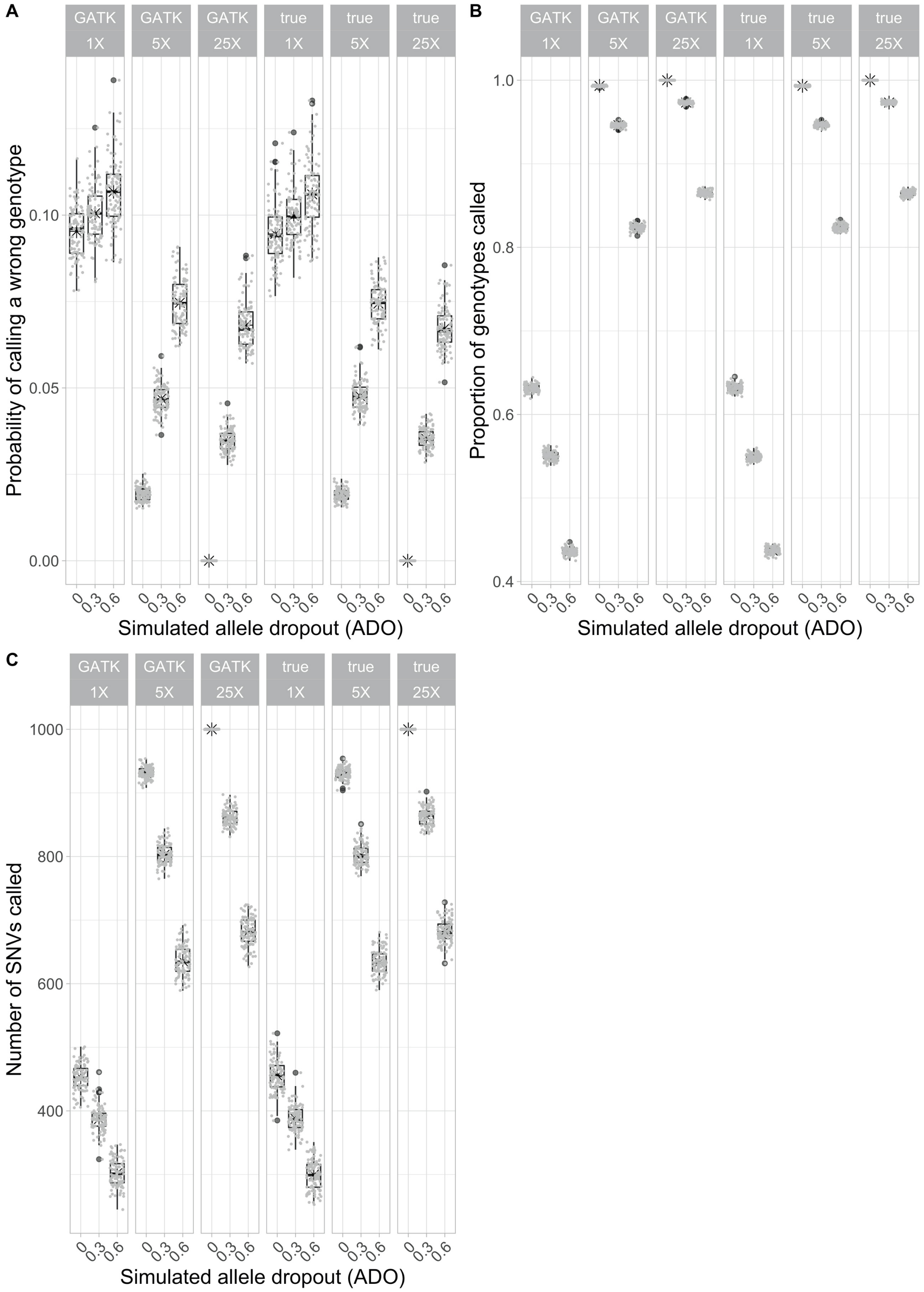


**FIG. S2.** Effect of single-cell allele dropout on genotype calling across sequencing coverages. *(A)* Probability that the maximum likelihood genotype is wrong, across variable and invariable sites. (*B*) Proportion of genotypes called. *(C)* Total number of single nucleotide variants (SNVs) called. *GATK* and *true* (GATK+ADO) are the likelihood models used for calling genotypes. 1X, 5X, and 25X are the sequencing coverages. Boxplots as in Figure S1.
